# Causality Patterns for Detecting Adverse Drug Reactions From Social Media: Text Mining Approach

**DOI:** 10.2196/publichealth.8214

**Published:** 2018-05-09

**Authors:** Danushka Bollegala, Simon Maskell, Richard Sloane, Joanna Hajne, Munir Pirmohamed

**Affiliations:** ^1^ Department of Computer Science University of Liverpool Liverpool United Kingdom; ^2^ Department of Translational Medicine University of Liverpool Liverpool United Kingdom

**Keywords:** machine learning, ADR detection, causality, lexical patterns, causality detection, support vector machines

## Abstract

**Background:**

Detecting adverse drug reactions (ADRs) is an important task that has direct implications for the use of that drug. If we can detect previously unknown ADRs as quickly as possible, then this information can be provided to the regulators, pharmaceutical companies, and health care organizations, thereby potentially reducing drug-related morbidity and saving lives of many patients. A promising approach for detecting ADRs is to use social media platforms such as Twitter and Facebook. A high level of correlation between a drug name and an event may be an indication of a potential adverse reaction associated with that drug. Although numerous association measures have been proposed by the signal detection community for identifying ADRs, these measures are limited in that they detect correlations but often ignore causality.

**Objective:**

This study aimed to propose a causality measure that can detect an adverse reaction that is caused by a drug rather than merely being a correlated signal.

**Methods:**

To the best of our knowledge, this was the first causality-sensitive approach for detecting ADRs from social media. Specifically, the relationship between a drug and an event was represented using a set of automatically extracted lexical patterns. We then learned the weights for the extracted lexical patterns that indicate their reliability for expressing an adverse reaction of a given drug.

**Results:**

Our proposed method obtains an ADR detection accuracy of 74% on a large-scale manually annotated dataset of tweets, covering a standard set of drugs and adverse reactions.

**Conclusions:**

By using lexical patterns, we can accurately detect the causality between drugs and adverse reaction–related events.

## Introduction

### Background

An adverse drug reaction (ADR) is defined as “an appreciably harmful or unpleasant reaction, resulting from an intervention related to the use of a medicinal product, which predicts hazard from future administration and warrants prevention or specific treatment, alternation of the dosage regimen, or withdrawal of the product” [1-4]. It is estimated that approximately 2 million patients in the United States are affected each year by serious ADRs, resulting in roughly 100,000 fatalities [5]. In fact, ADRs are the fourth leading cause of death in the United States, following cancer and heart diseases [6]. Treating patients who develop ADRs results in significant health costs to nations throughout the world. For example, it has been estimated that US $136 billion is spent each year on treatments related to ADRs in the United States [7,8].

In an ideal world, all adverse reactions associated with a drug need to be detected before marketing, and the drug label modified accordingly. However, this is not feasible due to several reasons. First, the number of human subjects participating in a clinical trial of a premarketed drug is often small, which limits the statistical power to detect ADRs, particularly those which may be uncommon. In fact, rare ADRs are usually not detected during the premarketing phases of drug development. Second, as many of the clinical trials are short-lasting, ADRs which are delayed will not be detected. Third, some ADRs show up only when a drug is being taken together with other drugs, leading to an adverse drug-drug interaction. Considering that the number of combinations of drugs is potentially large, it is impractical to test for all of the possible combinations during a clinical trial. Fourth, *drug repurposing* [9]—the practice of off-label usage of drugs for treating diseases for which they were not originally intended—could lead to unforeseen ADRs.

Due to these challenges in detecting ADRs during the premarketing phase, identification of ADRs in the postmarketing phase remains hugely important. The cornerstone of postmarketing pharmacovigilance remains the spontaneous reporting schemes such as the Yellow Card Scheme [10] in the United Kingdom and the MedWatch system [11] in the United States. Such schemes allow hospitals, medical practitioners, and patients to report ADRs. Unfortunately, the reporting rates are generally poor. For example, only 10% of serious ADRs and 2% to 4% of nonserious ADRs are reported [12].

Although patients experience ADRs, they may be reluctant to report their experiences through official reporting systems for various reasons. For example, patients might be unfamiliar with or unaware of the ADR reporting schemes, or might find it difficult to understand the terminology used in the forms, or might not be aware of the importance of reporting ADRs. Even when ADRs have been reported via such spontaneous reporting systems, the time required from the first report to any regulatory action may be long, which is problematical in protecting public health from iatrogenic conditions.

An alternative approach for detecting ADRs in a timely manner on a larger scale is to use social media. Social media platforms such as Twitter [13], Facebook [14], Instagram [15], and Pinterest [16] have been used extensively for market analysis of various products. Social media provides a convenient and direct access to consumers’ opinions about the products and services they use. In comparison with a clinical study, which inevitably is limited to a small number of participants, in social media we can access comments from a massive number of diverse groups of people. Due to its potential value, the pharmacovigilance community has already started to exploit social media as a potential reporting tool for obtaining information about ADRs [17]. For example, the WEB-RADR [18] project funded by the Innovative Medicines Initiative was funded to evaluate the usefulness of social media as a reporting tool for ADRs.

However, compared with spontaneous reporting systems where patients or health care practitioners explicitly report ADRs, detecting ADRs from social media poses several challenges. Because social media is not perceived by most patients as an official reporting tool for ADRs, a drug and its associated ADRs might not be completely expressed in a single social media post. This issue is further aggravated by the limitations imposed on the length of a post in social media platforms. For example, in Twitter, a single post (aka a *tweet*) is limited to a maximum of 140 characters. Even in social media platforms where such limitations do not exist, such as Facebook, the users might not always provide comprehensive reports containing all the information that would normally be completed on a Yellow Card. Furthermore, social media users often interact with social media platforms through specialized apps on mobile devices such as mobile phones, which do not possess physical keyboards that facilitate the entering of longer texts.

In addition to the brevity and incompleteness of social media posts as a medium for reporting ADRs, the reliability of the information expressed through social media is also a concern. It is often difficult to authenticate the information disseminated through social media. For example, in Twitter, the same user can create multiple accounts under different names including aliases. False information might be expressed intentionally or unintentionally in social media, which makes it difficult to verify the information extracted from social media. Unlike in the Yellow Card system, where it is possible to contact a reporter to obtain further information, in social media it is difficult to obtain additional information from users due to anonymity and privacy settings. All of these challenges introduce various levels of noise to ADR signal that can be captured from social media. Consequently, methods that detect ADRs from social media need to overcome these challenges.

An approach for detecting significant signals indicating adverse reactions to drugs in social media is to measure the correlation between a drug and an event. If many social media posts or users mention a drug and an event, then the likelihood that the drug causes an adverse reaction increases. Indeed, numerous measures have been proposed in previous work to measure the degree of association between a drug and an adverse reaction [19-26]. Although co-occurrence measures do not completely solve all of the above-mentioned challenges of using social media, they provide a practical and a highly scalable mechanism for detecting ADRs from social media.

A fundamental drawback of co-occurrence-based approaches for detecting ADRs is that they ignore the context in which a drug and an ADR co-occur in social media. Co-occurrence *does not* always indicate causality. Although a drug and an event that could suggest an ADR might be mentioned frequently in social media, the co-occurrence may be because the drug is used as a remedy for that symptom. Moreover, the drug may have been taken by 1 person, but the social media post mentions the ADR in a different person. However, the context in which a drug and an ADR co-occur can provide useful clues that can be used to separate causality from co-occurrence.

**Figure 1:**
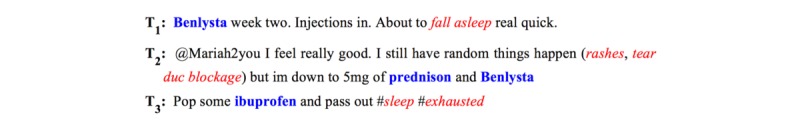
Three tweets mentioning a drug (shown in blue boldface fonts) and symptoms (shown in red italic font).

To illustrate the usefulness of contextual information for ADR detection, consider the 3 tweets shown in Figure 1. T_1_ is suggestive of an association with a drug and a potential adverse reaction. T_2_ may reflect that the patient’s disease improving or that an ADR occurred but is waning following dose reduction. T_3_ is unlikely to be an ADR; Ibuprofen is being taken by this patient to potentially relieve the pain and have some sleep. These examples show that there are useful hints we can extract from the tweets such as *about to (feel an ADR)*, *I still have (ADRs)* that we can use to evaluate the causality relationship between a mentioned drug and an adverse reaction.

Why is solving this problem critical for systems that attempt to extract ADRs from social media? The standard practice in the pharmacovigilance community for detecting ADRs from patient reports is to apply disproportionality measures that consider *only* co-occurrence (and occurrence) counts. Unfortunately, disproportionality measures by design are agnostic to the linguistic context in social media and are therefore unable to utilize the clues that appear in social media to determine whether an ADR is truly caused by the drug. However, given a tweet containing a drug and a potential adverse reaction, if we can first develop a classifier that predicts whether this tweet is describing a causality relationship, we then can use disproportionality measures on the tweets that are identified as positive by the classifier for further analysis. This preprocessing step is likely to improve the accuracy of the ADR detection process. Moreover, given the noise and the low level of reliability in social media as opposed to patient reports in spontaneous reporting schemes, it is vital that we perform some form of preprocessing to guarantee the reliability of the identified ADRs.

In this paper we, therefore, consider the following problem: given a tweet *T* containing a drug *D* and an ADR *A*, whether *T* describes an instance where *A* is caused by *D*, as opposed to *A* and *D* co-occurring for a different reason (or randomly without any particular relation between *A* and *D*). Our experimental results show that the proposed method statistically significantly outperforms several baseline methods, demonstrating its ability to detect causality between drugs and ADRs in social media.

### Related Work

The number of co-occurrences between a drug and an ADR can be used as a signal for detecting ADRs associated with drugs. Various measures have been proposed in the literature that evaluate the statistical significance of disproportionally large co-occurrences between a drug and an ADR. These include *Multiitem* Gamma Poisson Shrinker [24,26-28], Regression-Adjusted Gamma Poisson Shrinker [23], Bayesian Confidence Propagation Neural Network (BCPNN) [20-22], Proportional Reporting Rate [19,28], and Reporting Odds Ratio [19,28]. Each of these algorithms uses a different measure of disproportionality between the signal and its background. Information component is applied in BCPNN, whereas empirical Bayes geometric mean is implemented in all variants of the Gamma Poisson Shrinker algorithm. Each of the measures gives a specific score, which is based on the number of reports including the drug or the event of interest. These count-based methods are collectively referred to as *disproportionality measures*.

In contrast to these disproportionality measures that use only co-occurrence statistics for determining whether there is a positive association between a drug and an event, in this paper, we propose a method that uses the contextual information extracted from social media posts to learn a classifier that determines whether there is a causality relation between a drug and an ADR. Detecting causality between events from natural language texts has been studied in the context of discourse analysis [29,30] and textual entailment [31,32]. In discourse analysis, a discourse structure for a given text is created, showing the various discourse relationships such as causality, negation, and evidence. For example, in Rhetorical Structure Theory [33], a text is represented by a discourse tree where the nodes correspond to sentences or clauses referred to as elementary discourse units (EDUs), and the edges that link those textual nodes represent various discourse relations that exist between 2 EDUs. Supervised methods that require manually annotated discourse trees [34] as well as unsupervised methods that use discourse cues [35] and topic models [36] have been proposed for detecting discourse relations.

The problem of determining whether a particular semantic relation exists between 2 given entities in a text is a well-studied problem in the natural language processing (NLP) community. The context in which 2 entities co-occur provides useful clues for determining the semantic relation that exists between those entities. Various types of features have been extracted from co-occurring contexts for this purpose. For example, Cullotta and Sorensen [37] proposed tree kernels that use dependency trees. Dependency paths and the dependency relations over those paths are used as features in the kernel. Agichtein and Gravano [38] used a large set of automatically extracted surface-level lexical patterns for extracting entities and relations from large text collections.

To address the limitations of co-occurrence-based approaches, several prior studies have used contextual information [39]. Nikfarjam et al [40] annotated tweets for ADRs, beneficial effects, and indications and used those tweets to train a Conditional Random Field. They use contextual clues from tweets and word embeddings as features. Their problem setting is different from ours in the sense that we do not attempt to detect/extract ADRs or drug names from tweets but are only interested in determining whether the mentioned ADR is indeed relevant to the mentioned drug. A tweet can mention an ADR and a drug, but the ADR might not necessarily be related to the ADR. Huynh et al [41] proposed multiple deep learning models by concatenating convolutional neural network (CNN) and recurrent neural network architectures to build ADR classifiers. Specifically, given a sentence, they would like to create a binary classifier that predicts whether the sentence contains an ADR or otherwise. Their experimental results show CNNs to be the best for ADR detection. This observation is in agreement with broader text classification tasks in NLP where CNNs have reported the state-of-the-art performance [42]. However, one issue when using CNNs for ADR detection is the lack of labeled training instances, such as annotated tweets. This problem is further aggravated if we must learn embeddings of novel drugs or rare ADRs as part of the classifier training.

To overcome this problem, Lee et al [43] proposed a semisupervised CNN that can be pretrained using unlabeled data for learning phrase embeddings. Bidirectional Long Short-Term Memory (bi-LSTM) units were used [44] to tag ADRs and indicators in tweets. A small collection of 841 tweets was manually annotated by 2 annotators for this purpose. Pretrained word embeddings using skip-gram on 400 million tweets are used to initialize the bi-LSTM’s word representations. This setting is different to what we study in this paper because we do not aim to tag ADRs and indicators in a tweet but to determine whether a tweet that mentions an ADR and a drug indicator describes an ADR event related to the drug mentioned in the tweet.

## Methods

### Overview

In this section, we presented our proposed method for detecting the causality between a drug and an event. First, in the section on “Problem Definition”, we formally define the problem of causality detection between a drug and an event from social media posts. Next, we explain techniques for aggregating social media posts related to drugs and events. Next, we explain the method we use for extracting various lexical patterns that described the relationship between a drug and an event in social media posts. Finally, we present a machine learning approach that uses a manually annotated dataset containing social media posts as to whether they are describing a relationship between a drug and an adverse reaction for learning the reliability of the lexical patterns we extract. We have not assumed any specific properties or meta-data available in a particular type of social media platform such as *retweets*, *favorites* in Twitter, or *likes* or *comments* in Facebook. Although such platform-specific metadata can provide useful features for a machine learning algorithm, such metadata are not universally available across all social media platforms or cannot be retrieved due to privacy settings. The fact that the proposed method does not rely on such metadata was attractive because it made our proposed method applicable to a wide range of social media posts and does not limit it to a particular platform.

### Problem Definition

Let us consider a social media post *T*, which explicitly mentions a drug *D* and an adverse reaction *R*. We model the problem of detecting causality between *D* and *R* in *T* as a binary classification problem where we would like to learn a binary classifier *h* (*T*, *D, R; w*) parametrized by a *d*-dimensional real-valued weight vector w ∈ℝ^d^ as shown in equation 1:

(1) If
*T* mentions that
*D* causes
*R*, then
*h*(
*T, D, R;w*)=1 and otherwise it is 0.

Here, we assume that the social media post *T* is already given to us and the drug and adverse reaction have already been detected in *T*. Detecting drug names can be done by matching against precompiled drug name lists (gazetteers) or using Named Entity Recognition [45]. A particular challenge when matching drug names in social media is that the drug names mentioned in social media might not necessarily match against the drug names listed in pharmacology databases [17]. The same drug is often sold under different labels by different manufacturers, and the label names continuously change, which makes it difficult to track a particular drug over time in social media. Similar challenges are encountered when matching ADRs in texts. Although the MedDRA [46] hierarchy assigns unique codes to preferred terms (PTs) that describe various ADRs such as “oropharyngeal swelling” or “systemic inflammatory response syndrome,” such terms are used rarely by the majority of the social media users who might not necessarily be familiar with the MedDRA code names [47]. Although we acknowledge the challenges in detecting mentions of drug names and adverse reactions, we consider it to be beyond the scope of this paper, which focuses on a signal detection problem.

### Social Media Aggregation

Although the problem definition described in Section 3.1 assumes that we are already provided with a set of social media posts, obtaining a large collection of social media posts relevant to drugs and events can be challenging for several reasons.

The vast majority of social media posts are not relevant to drugs or ADRs. One effective method for filtering out such irrelevant social media posts is to use the keyword-based filtering functionalities provided by the major social media application programming interfaces (APIs). As a specific example of such an API, we discuss the use of Twitter streaming API [48]. The Twitter streaming API allows registration of a set of keywords, and if there are any tweets that contain at least one of those keywords, then the corresponding tweet will be filtered and sent to the querying user. In our case, we used drug names and PTs (and their lexical variants) as keywords to filter the relevant tweets. Moreover, the streaming API also enabled us to limit the tweets to a particular geographical area or a language, which is useful if we want to monitor drugs that are specifically used in a particular country or a region.

Twitter’s streaming API allowed us to aggregate tweets from 2 main types of data streams: *public streams* and *user streams*. Public streams are publicly available tweets by a specific group of users or on a topic. Hash tags in twitter are useful for streaming such public tweets on a particular topic. For example, by including the hash tag *#epilepsy*, we can retrieve tweets that are relevant to epilepsy. On the other hand, user streams allow us to obtain tweets from a single twitter user, containing roughly all of the data corresponding with that user’s view (timeline) on Twitter. Despite the used aggressive filtering, streaming API returned a large number of tweets. Therefore, we stored the filtered tweets in a MongoDB [49] database in JavaScript Object Notation format for efficient retrieval.

### Lexical Pattern Extraction

**Figure 2:**
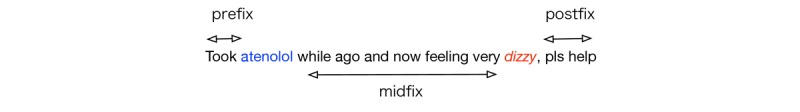
Extracting lexical patterns from a tweet that describes an adverse reaction (dizziness) caused by a drug (Atenolol). The tweet is split into 3 parts—prefix, midfix, and postfix, and various lexical patterns are extracted from each part. See text for the details of the pattern extraction method. Best viewed in color.

To represent the relationship between a drug and an ADR in a tweet, we extracted lexical patterns from the tweet. Let us illustrate the lexical pattern extraction process using the example tweet shown in Figure 2. We first identified the drug and event in the tweet and split the tweet into 3 parts. The part from the beginning of the tweet to the first-mentioned entity (either the drug or event) is named as the *prefix*, the part from the first-mentioned entity to the second-mentioned entity is named as the *midfix*, and the part from the second-mentioned entity to the end of the tweet is named as the *postfix*. Prior work on information extraction has shown that, in English, the midfix provides useful clues related to the relationship between 2 entities that co-occur in some context [50,51]. Indeed, from the example shown in Figure 2, we see that words such as *feeling* that appear in the midfix indicate that this twitter user is experiencing a side effect from the drug. However, it has also been shown that prefix and postfix terms also provide useful information when determining the relationship between 2 entities. For example, we see that the word *took* that appears in the prefix in the tweet (Figure 2), indicating that this twitter user has indeed taken this drug and not simply reporting an adverse reaction experienced by a different person. Such information is useful to estimate the reliability of the relationships mentioned in social media, which can often be noisy and unreliable. Therefore, in this work, we use all prefix, midfix, and postfix sections in tweets for extracting lexical patterns. We experimentally evaluate the significance of prefix, midfix, and postfix for ADR detection later in Section 4.

We extracted skip-grams from prefix, midfix, and postfix separately as lexical patterns for representing the relationship between a drug and an event. A skip-gram is an extension of *n*-gram. Unlike, *n*-grams that require us to consider all consecutive *n* words in a sequence, skip-grams allow us to generalize the *n*-gram patterns by skipping one or more words in a sequence. For example, trigram (*n*=3) lexical patterns extracted from the midfix shown in Figure 2 would be *while ago and*, *ago and now*, *and now feeling*, *now feeling very*.

On the other hand, skip-gram patterns also let us match any word (indicated by the wildcard “*”) in an *n*-gram pattern. For example, the skip-gram pattern * ago, which is a generalization of the bigram pattern *while ago* will match various other time indicators such as *hours ago*, *days ago*, and *months ago*. Unlike, *n*-gram patterns that might not match exactly in numerous other tweets, skip-gram patterns flexibly match different tweets, thereby leading to a dense feature space. More importantly, skip-gram patterns subsume *n*-gram patterns. Therefore, all tweets that can be represented using *n*-gram patterns can be matched by the corresponding skip-gram patterns.

Considering the fragmented, ungrammatical, and misspelled texts frequently encountered in social media, skip-gram lexical patterns provide a robust and flexible feature representation. Moreover, extracting skip-grams is computationally efficient compared with, for example, part-of-speech tagging or dependency parsing social media, considering the volume of the texts we must process. Note that the drug name or the event are *not* part of the skip-gram lexical patterns. In other words, we replace the drug name and event, respectively, by place holder variables D and R. This is important because we would like to generate patterns that not only match the existing drugs and adverse reactions but can generalize to future drugs and their (currently unknown) adverse reactions. In our experiments, we use skip-gram lexical patterns for *n*=1, 2, and 3 and allowed a maximum of 1 wildcard in a pattern.

### Learning Pattern Weights

We built a binary classifier that could predict whether an event *R* mentioned in a tweet *T* alongside a drug *D* was actually related to *D*. As explained later in Section 4.1, we used a manually annotated collection of tweets where each tweet contained a drug and an event, and a human annotator annotates whether the mentioned ADR is relevant to the drug (positively labeled instance) or otherwise (negatively labeled instance). We represent a tuple (*T*, *D*, *R*) using a feature vector f (*T, D, R*)∈ℝ^d^, where each dimension corresponds to a particular skip-gram lexical pattern, which we extracted following the procedure described in Section 3.3. The value of the *i*-th dimension in the feature vector is set to 1 if the skip-gram lexical pattern I_i_*l*_i_, *D*, *R*) is represented by a boolean-valued feature vector over the set of skip-gram lexical patterns we extracted from all of the training instances. Using the above notation, let us denote this training dataset by *D*_train_={(f (*T*_n_*, D*_n_*, R*_n_), y_n_)}^N^_n=1_. Here, (*T*_n_*, D*_n_*, R*_n_) indicates the *n*-th training instance out of *N* total instances in the dataset, and y_n_∈{−1,+1}.

Unfortunately, not all skip-gram lexical patterns are equally important when determining whether there exists a relationship between a drug and an event. For example, in Figure 2, the pattern *while ago* can appear in various contexts, not necessarily in the context where an adverse reaction is described. Therefore, we assigned some form of a *confidence weight* to each skip-gram pattern before we used those patterns to make a decision about the relationship between a drug and an event. For this purpose, we assigned a weight *w* and *R* in *T* using the linear binary classifier given by equation 2:

(2) h(T, D, R; w)=sgn(w
^T^f(T,D,R))

Here, w**∈**ℝ^d^ is a *d*-dimensional real-valued weight vector where the *i*-th dimension represents the confidence weight w_i_ and the sign function sgn is defined in equation 3:

(3) sgn(x)=1 if x>0 and −1 otherwise

**Figure 3:**
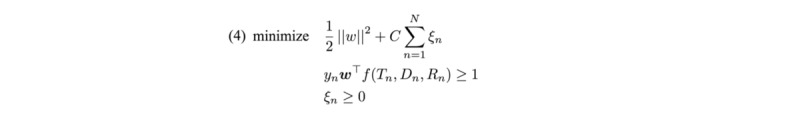
Support vector machine—optimization problem.

Given the training dataset *D*_train_, our goal was to learn *w* such that it can be used in equation 2 to predict whether the *R* mentioned in a *T* with *D* was indeed related to *D*. For this purpose, we used linear kernel support vector machines (SVMs) [52] with slack variables ξ_n_≥0 noise in training instances. Given the scale of the annotation task, it is unavoidable that some of the instances will be incorrectly labeled by the human annotators, introducing some labeling noise to the training dataset. Second, slack variables can shift some of the training instances closer to the decision hyperplane, thereby artificially making the dataset to be linearly separable.

Although nonlinear kernels such as polynomial, radial basis function (RBF), or sigmoid can be used with SVMs, we limited our analysis to linear kernels for the following reason. Under the linear kernel, the weight associated with a particular feature can be seen as the influence imparted by that feature on the classification decision. This property is useful because we can identify the most discriminative lexical patterns that indicate a positive association between a drug and an event. We can use such lexical patterns, for example, to create extraction rules in the form of regular expressions to extract adverse reactions of drugs from social media. Because we are using a linear classifier in this work, it is important to handle the instances that violate the decision hyperplane using slack variables.

The joint learning of slack variables and weights can be formulated as the constrained convex optimization problem given by equation 4 in Figure 3.

Here, *C*>0, cost factor, is a hyperparameter that determines how much penalty we assigned to margin violations. The optimization problem given in equation 4 can be converted into a quadratic programming problem by introducing Lagrange multipliers. Efficient implementations that scale well to large datasets with millions of instances and features have been proposed [53].

Once we have obtained the weights w_i_, equation 2 can be used to predict the relationship between *D* and *R* in *T*.

## Results

We trained and evaluated the proposed method using a manually annotated dataset. The details of the dataset are presented in Section 4.1. Next, to evaluate the proposed method we compared it with several baseline methods. The baseline methods and their performances are described in Section 4.

To create a training and testing dataset for our task, we manually annotated a set of social media posts collected from the Twitter and Facebook between August 2015 and October 2015. Using the social media aggregation techniques described in Section 3.2, we filtered social media posts that contained a single mention of a drug and an event. The number of tweets that contain both a PT and a drug name was 94,890.

We then asked a group of annotators, who are familiar with ADRs of drugs, to annotate whether the event mentioned in the social media post is caused by the drug mentioned in the same post (a positively labeled instance) or otherwise (a negatively labeled instance).

The final annotated dataset contained 44,809 positively labeled instances and 50,081 negatively labeled instances. We perform 5-fold cross-validation on this dataset, selecting 80% of the positive and negative instances in each fold as training data, and the remainder as the testing data. In addition to the above-mentioned social media posts, we set aside 1000 positively and 1000 negatively labeled social media posts as developmental data, for tuning the hyperparameter *C*. In total, we extracted 168,663 skip-gram patterns from this dataset. We used classification accuracy defined by equation 5 as the evaluation measure:

(5) Classification Accuracy=Total number of correctly predicted instances/Total number of instances in the dataset

## Discussion

### Baselines

We compared the proposed method with several baseline methods using the classification accuracy on the testing data as shown in Table 1. Next, we describe the different methods compared in Table 1.

#### Majority Baseline

Note that our training and test datasets were unbalanced in the sense that we have more negatively labeled instances than positively labeled instances. This situation is natural, given that most social media posts might not necessarily describe an adverse reaction of a drug even though it mentioned both the drug and an event. The training and test datasets we used in our evaluations closely simulate this situation. However, if a dataset is unbalanced, then by simply predicting the majority class (in our case this is the negative label) can still result in classification accuracies greater than 50%. The majority baseline shows the level of performance that was obtained by such a majority classifier.

#### Bag-of-Words Classifier

Our proposed method used skip-gram patterns for representing social media posts. An alternative approach would be to ignore the word order in the text and represent a text using the set of words contained in it. Specifically, we would represent each text by a binary-valued feature vector where the feature values for the unigrams that appear in the text are set to 1, and 0 otherwise. We then trained a binary SVM classifier with a linear kernel. By comparing against the bag-of-words (BOW) classifier, we can empirically evaluate the usefulness of the proposed skip-gram lexical patterns.

#### Prefix Only

This is a scaled-down version of the proposed method that used skip-gram patterns extracted only from the prefix. By evaluating against the prefix only baseline, we evaluated the importance of the information contained in the prefix. There are 50,021 prefix skip-gram patterns in total.

#### Midfix Only

This is a scaled-down version of the proposed method that uses skip-gram patterns extracted only from the midfix. By evaluating against the midfix only baseline, we evaluated the importance of the information contained in the midfix. There are 53,057 midfix skip-gram patterns in total.

#### Postfix Only

This is a scaled-down version of the proposed method that uses skip-gram patterns extracted only from the postfix. By evaluating against the postfix only baseline, we evaluated the importance of the information contained in the postfix. There are 65,585 postfix skip-gram patterns in total.

#### Prefix+Midfix

In this baseline method, we used both prefix and midfix for extracting skip-gram patterns. This baseline demonstrates the effectiveness of combining contextual information from both the prefix and the midfix.

#### Prefix+Postfix

In this baseline method, we used both prefix and postfix for extracting skip-gram patterns. This baseline demonstrates the effectiveness of combining contextual information from both the prefix and the postfix.

#### Midfix+Postfix

In this baseline method, we use both midfix and postfix for extracting skip-gram patterns. This baseline demonstrates the effectiveness of combining contextual information from the midfix and the postfix.

#### Convolutional Neural Network

We use the state-of-the-art short text classification method proposed by Kim [42] to train an ADR classifier. Each word in a tweet is represented using 128 dimensional word embeddings, where each dimension is randomly sampled from a uniform distribution in range (−1,1). The word embeddings are concatenated to represent a tweet. Next, a one-dimensional CNN with a stride size of 3 tokens and a max pooling layer is applied to create a fixed 20-dimensional tweet representation. We use Adaptive Subgradient Method [54] for optimization with initial learning rate set to 0.01 and the maximum number of iterations set to 1000. Finally, logistic sigmoid unit is used to produce a binary classifier.

#### Proposed Method

This is the method proposed in this paper. We use prefix, midfix, and postfix for extracting skip-gram patterns.

Using the development data, we found the cost parameter *C* for each setting. For the BOW classifier, the optimal *C* value was found to be 0.01, whereas for all the variants of the proposed method, it was 1.0.

The classification accuracies obtained for the 5-fold cross-validation task for the above-mentioned methods are shown in Table 1. From Table 1, we see that the majority baseline achieves an accuracy of 63.19%. Our task here is binary classification, and to compute confidence intervals for accuracies, we must compute binomial confidence intervals. There are several ways to compute this and one approach is the use of Clopper-Pearson confidence intervals [55]. By using confidence intervals, we can easily compare the statistical significance between methods, without having to conduct numerous pairwise comparisons between different methods. We compared all other methods against the accuracy reported by the majority baseline using Clopper-Pearson confidence intervals (*P*<.001) to test for statistical significance, which is (61.70,65.65). Statistically significant accuracies over the majority baseline are indicated by a superscripted letter a in Table 1.

From Table 1, we see that the best performance is obtained by the proposed method using the skip-gram patterns extracted from all prefix, midfix, and suffix contexts. A skip-gram pattern is an extension of n-gram patterns. Unlike n-gram patterns that must contain consecutive tokens, skip-gram patterns can skip one or more tokens when representing a subsequence. Among the different context types, we see that midfix performs best, whereas prefix and postfix perform relatively equally. This result is in agreement with prior work on information extraction for English, where midfix has been found to be useful. However, to the best of our knowledge, such an analysis has not yet been conducted for ADR extraction. Interestingly, we see that by adding the midfix to prefix and postfix we always perform better than if we had used only prefix or postfix. The proposed method uses all 3 contexts and obtains the best performance among the methods compared in Table 1. In particular, the performance reported by the proposed method is statistically significant over both the majority baseline and the BOW classifier. We see that the CNN-based ADR classifier is performing at the same level as the BOW classifier. Compared with the typical sentence classification datasets used to train such deep learning methods, our twitter dataset is significantly smaller, and this lack of data might have resulted in CNN-based ADR classifier to perform poorly in our experiments.

**Figure 4:**
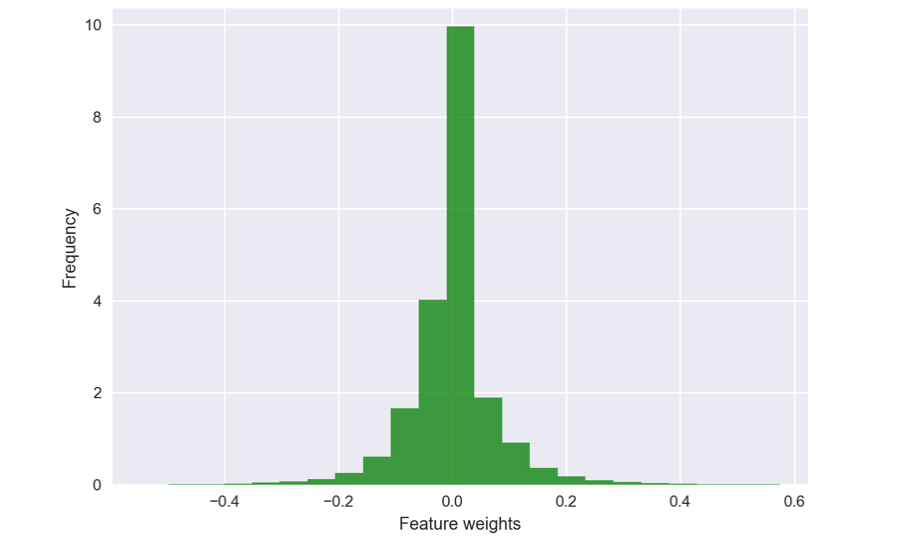
Histogram of the weights of the features learned by the support vector machine (SVM) classifier.

To gain further insights into the skip-gram patterns that are identified by the classifier to be useful for predicting whether there is a positive relationship between a drug and an event in a tweet, we plot the histogram of the feature weights in Figure 4. From Figure 4, we see that the majority of patterns have their weights close to zero, and an almost identical spread in positive and negative directions centered around zero. We counted 60,430 patterns to have weights exactly set to zero, meaning that approximately 35.83% (60,430/168,663) of patterns are found to be uninformative by the classifier. A randomly selected subset of zero-weighted patterns is shown in Table 1. Although there is a large number of patterns used as features, patterns that are not discriminative for the purpose of detecting ADRs are effectively pruned out by the SVM by assigning lower weights as shown in Table 2. Therefore, even if we have a comparatively larger feature space to the number of training instances, this does not necessarily result in overfitting.

We list the top-ranked positively weighted and negatively weighted skip-gram patterns in Table 3. From Table 3, we see that skip-gram patterns that describe a positive relationship between a drug and an ADR are correctly identified by the proposed method. For example, the *P+took+too* indicates that the user has actually took the drug. Moreover, we see many negations in the top-ranked negatively weighted patterns. Such clues could be used in several ways. First, we can use these clues as keywords for filtering social media posts that describe a potential positive relationship between drugs and ADRs. For example, we could run disproportionality-based signal detection methods using the disproportionality counts obtained from those filtered social media posts, thereby increasing the reliability of the detection. Second, these clues could be used to develop extraction patterns/templates that can be used for matching and extracting previously unknown ADRs for novel or existing drugs.

**Table 1:** Classification accuracy of different baselines and the proposed method.

Method	Classification accuracy
Majority baseline	63.19
Bag-of-words classifier	69.31^a^
Convolutional neural network	69.26^a^
Prefix only	66.41^a^
Midfix only	72.78^a^
Postfix only	68.08^a^
Prefix+midfix	74.72^a^
Prefix+postfix	71.07^a^
Midfix+postfix	77.10^a^
Proposed method	77.70^a^

^a^Statistically significant values.

**Table 2:** A randomly selected sample of features with zero weights.

Prefix patterns	Midfix patterns	Postfix patterns
P+trip+i	M+bad+idea	S+over
P+news+:	M+a+breakfast	S+12+hours
P+dat+lean	M+if+school	S+conquest
P+@rroddger	M+medica_authorities	S+please
P+fussiness+no	M+convicted+i	S+bad!

**Table 3:** Top-ranked positively (left 2 columns) and negatively (right 2 columns) weighted features (skip-gram patterns) by the support vector machine.

Feature	Weight	Feature	Weight
S^c^+als	1.2096	M+commercial	−1.2304
M^b^+induced	1.1314	P+hate+being	−1.0398
P^a^+oh+no	1.0683	P+I’m+definitely	−1.0000
M+^d^stinks	1.0000	P+clumsiness	−1.0000
S+.+wooh	1.0000	P+hospitalization	−1.000
M+never+work	1.0000	S+lol+fml	−0.9674
P+high+off	0.9006	S+wopps	−0.9035
P+took+too	0.8449	P+rt+xanaaxhadme	−0.8067
M+was+supposed	0.8378	P+don’t+think	−0.7721

^a^P: prefix skip-gram patterns.

^b^M: midfix skip-gram patterns.

^c^S: postfix skip-gram patterns.

^d^For bigrams, we have used “+” to separate the constituent unigrams.

### Conclusions

We proposed a novel signal detection problem where given a social media post *T* that contains a drug *D* and an event *R*, we would like to determine whether *R* is related to *D*, or otherwise. We have then proposed a method to solve this signal detection problem utilizing the lexical contextual information in *T*. Specifically, we extracted skip-gram patterns from the prefix, midfix, and suffix in *T*, and trained a binary SVM using a manually labeled training dataset. Our results show that the proposed method significantly outperformed the majority baseline and a BOW classifier. Moreover, we showed that the discriminative patterns were ranked at the top by the trained classifier. In the future, we plan to use the automatically extracted patterns to develop an ADR extraction method for previously unknown adverse reactions of drugs from social media.

## Abbreviations

ADRs: adverse drug reactions
API: application programming interface
BCPNN: Bayesian Confidence Propagation Neural Network
Bi-LSTM: Bidirectional Long Short-Term Memory
BOW: bag of words
CNN: convolutional neural network
EDUs: elementary discourse units
NLP: natural language processing
PTs: preferred terms
SVMs: support vector machines

## References

[1] Yang CC, Yang H, Jiang L, Zhang M (2012). Social media mining for drug safety signal detection.

[2] Edwards IR, Aronson JK (2000). Adverse drug reactions: definitions, diagnosis, and management. Lancet.

[3] World Health Organization (1969). International drug monitoring. The role of the hospital. World Health Organ Tech Rep Ser.

[4] Lee A (2006). Adverse Drug Reactions.

[5] Leaman R, Wojtulewicz L, Sullivan R, Skariah A, Yang J, Gonzalez G (2010). Towards internet-age phramacovigilance: extracting adverse drug reactions from user posts to health-related social networks.

[6] Giacomini KM, Krauss RM, Roden DM, Eichelbaum M, Hayden MR, Nakamura Y (2007). When good drugs go bad. Nature.

[7] Bates DW, Evans RS, Murff H, Stetson PD, Pizziferri L, Hripcsak G (2003). Detecting adverse events using information technology. J Am Med Inform Assoc.

[8] van der Hooft CS, Sturkenboom MC, van Grootheest K, Kingma HJ, H Ch Stricker B (2006). Adverse drug reaction-related hospitalisations. Drug Safety.

[9] Rastegar-Mojarad M, Liu H, Nambisan P (2016). Using social media data to identify potential candidates for drug repurposing: a feasibility study. JMIR Res Protoc.

[10] Yellow Card.

[11] Food and Drug Administration.

[12] Chaplin S (2006). The yellow card scheme? Why are GPs under-reporting?. Prescriber.

[13] Twitter.

[14] Facebook.

[15] Instagram.

[16] Pinterest.

[17] Sloane R, Osanlou O, Lewis D, Bollegala D, Maskell S, Pirmohamed M (2015). Social media and pharmacovigilance: a review of the opportunities and challenges. Br J Clin Pharmacol.

[18] WEB-RADR.

[19] Suling M, Pigeot I (2012). Signal detection and monitoring based on longitudinal healthcare data. Pharmaceutics.

[20] Norén GN, Bate A, Orre R, Edwards IR (2006). Extending the methods used to screen the WHO drug safety database towards analysis of complex associations and improved accuracy for rare events. Stat Med.

[21] Bate A, Lindquist M, Edwards IR, Orre R (2002). A data mining approach for signal detection and analysis. Drug Safety.

[22] Bate A, Lindquist M, Edwards IR, Olsson S, Orre R, Lansner A, De Freitas RM (1998). A Bayesian neural network method for adverse drug reaction signal generation. Eur J Clin Pharmacol.

[23] DuMouchel W, Harpaz R (2012). Regression-adjusted gps algorithm (RGPS). Oracle Health Sci.

[24] Ahmed I, Haramguru F, Fourrier-Reglat A, Thiessard F, Kreft-Jias C, Miremont-Salame G, Begaud B, Tubert-Bitter P (2009). Bayesian pharmacovigilance signal detection methods revisited in a multiple comparison setting. Stat Med.

[25] Fram DM, Almenoff JS, DuMouchel W (2003). Empirical Bayesian data mining for discovering patterns in post-marketing drug safety.

[26] DuMouchel W, Pregibon D (2001). Empirical bayes screening for multi-term associations.

[27] Dumouchel W (1999). Bayesian data mining in large frequency tables, with an application to the FDA spontaneous reporting system. Am Stat.

[28] Hauben M, Zhou X (2003). Quantitative Methods in Pharmacovigilance. Drug Safety.

[29] Do QX, Chan YS, Roth D (2011). Minimally supervised event causality identification.

[30] Radinsky K, Davidovich S, Markovitch S (2012). Learning causality for news events prediction.

[31] Androutsopoulos I, Malakasiotis P (2010). A survey of paraphrasing and textual entailment methods. J Artif Intell Res.

[32] Angel Ríos Gaona M, Gelbukh A, Bandyopadhay S (2010). Recognizing textual entailment with statistical methods.

[33] Mann WC, Thompson SA (1988). Rhetorical structure theory: toward a functional theory of text organization. Text Talk.

[34] duVerle DA, Prendinger H (2009). A novel discourse parser based on support vector machine classification.

[35] Marcu D, Echihabi A (2002). An unsupervised approach to recognizing discourse relations.

[36] Ó Séaghdha D, Teufel S (2014). Unsupervised learning of rhetorical structure with un-topic models. http://www.aclweb.org/anthology/C14-1002.

[37] Culotta A, Sorensen J (2004). Dependency Tree Kernels for Relation Extraction. http://aclweb.org/anthology/P/P04/P04-1054.pdf?CFID=28410093&CFTOKEN=4e679354be37e14d-B170E708-9B8A-B137-2D13DA2B3C10E01A.

[38] Agichtein E, Gravano L (2000). Snowball: extracting relations from large plain-text collections.

[39] Lardon J, Abdellaoui R, Bellet F, Asfari H, Souvignet J, Texier N, Jaulent MC, Beyens MN, Burgun A, Bousquet C (2015). Adverse drug reaction identification and extraction in social media: a scoping review. J Med Internet Res.

[40] Nikfarjam A, Sarker A, O'Connor K, Ginn R, Gonzalez G (2015). Pharmacovigilance from social media: mining adverse drug reaction mentions using sequence labeling with word embedding cluster features. J Am Med Inform Assoc.

[41] Huynh T, He Y, Willis A, Rueger S (2016). Adverse drug reaction classification with deep neural networks. http://www.aclweb.org/anthology/C16-1084.

[42] Kim Y (2014). Convolutional neural networks for sentence classification. http://www.aclweb.org/anthology/D14-1181.

[43] Lee K, Qadir A, Hasan S, Datla V, Prakash A, Liu J, Farri O (2017). Adverse Drug Event Detection in Tweets with Semi-Supervised Convolutional Neural Networks.

[44] Cocos A, Fiks AG, Masino AJ (2017). Deep learning for pharmacovigilance: recurrent neural network architectures for labeling adverse drug reactions in Twitter posts. J Am Med Inform Assoc.

[45] Segura-Bedmar I, Martínez P, Segura-Bedmar M (2008). Drug name recognition and classification in biomedical texts. A case study outlining approaches underpinning automated systems. Drug Discov Today.

[46] MedDRA.

[47] Limsopatham N, Collier N (2015). Adapting phrase-based machine translation to normalise medical terms in social media messages. https://aclanthology.info/pdf/D/D15/D15-1194.pdf.

[48] Twitter.

[49] MongoDB.

[50] Bollegala D, Matsuo Y, Ishizuka M (2009). A relational model of semantic similarity between words using automatically extracted lexical pattern clusters from the web.

[51] Bollegala D, Matsuo Y, Ishizuka M (2009). Measuring the similarity between implicit semantic relations from the web.

[52] Vapnik VN (1998). Statistical Learning Theory.

[53] Fan RE, Chang KW, Hsieh CJ, Wang XR, Lin CJ (2008). LIBLINEAR: A library for large linear classification. J Mach Learn Res.

[54] Duchi J, Hazan E, Singer Y (2011). Adaptive subgradient methods for online learning and stochastic optimization. J Mach Learn Res.

[55] Clopper CJ, Pearson ES (1934). The use of confidence or fiducial limits illustrated in the case of the binomial. Biometrika.

